# Proteomic Analysis of Chinook Salmon (*Oncorhynchus tshawytscha*) Ovarian Fluid

**DOI:** 10.1371/journal.pone.0104155

**Published:** 2014-08-04

**Authors:** Sheri L. Johnson, Marsha Villarroel, Patrice Rosengrave, Alan Carne, Torsten Kleffmann, P. Mark Lokman, Neil J. Gemmell

**Affiliations:** 1 Allan Wilson Centre for Molecular Ecology and Evolution and Department of Anatomy, University of Otago, Dunedin, New Zealand; 2 Department of Zoology, University of Otago, Dunedin, New Zealand; 3 Centre for Protein Research, Department of Biochemistry, University of Otago, Dunedin, New Zealand; Universitat de Barcelona, Spain

## Abstract

The ovarian, or coelomic, fluid that is released with the egg mass of many fishes is increasingly found to play an important role in several biological processes crucial for reproductive success. These include maintenance of oocyte fertility and developmental competence, prolonging of sperm motility, and enhancing sperm swimming speed. Here we examined if and how the proteome of chinook salmon (*Oncorhynchus tshawytscha*) ovarian fluid varied among females and then sought to examine the composition of this fluid. Ovarian fluid in chinook salmon was analyzed using 1D SDS PAGE and LC-MS/MS tryptic digest screened against Mascot and Sequest databases. We found marked differences in the number and concentrations of proteins in salmon ovarian fluid across different females. A total of 174 proteins were identified in ovarian fluid, 47 of which were represented by six or more peptides, belonging to one of six Gene Ontology pathways. The *response to chemical stimulus* and *response to hypoxia* pathways were best represented, accounting for 26 of the 174 proteins. The current data set provides a resource that furthers our understanding of those factors that influence successful egg production and fertilisation in salmonids and other species.

## Introduction

Ovarian fluid surrounds the eggs of many externally fertilising female fish and is released with the spawned eggs into fresh or salt water [1]. Due to the viscosity of ovarian fluid, the fluid encapsulates the eggs during spawning [2]. Concentrations of ovarian fluid are likely to be high close to the egg outer membrane, peaking inside the egg micropyle, where they may reach close to 100%, through which sperm must swim to achieve fertilisation [3], [4]. Hence, both sperm activation and egg fertilisation likely occur in the presence of ovarian fluid in many fish species [5].

In salmonids, ovarian fluid (also known as coelomic fluid) is derived from secretory epithelia in the ovaries and filtered blood plasma [6]. Ovarian fluid makes up 10–30% of the volume of the spawned egg mass [2], [7], and unlike most other teleost fish, in which the lumen of the ovary is continuous with the oviduct (cystovarian; [8], [9], in salmonids this fluid bathes ovulated eggs while they are in the body cavity (gymnovarian; [8]–[10]) and has continuous interchange with the circulating plasma. Thus, in salmonids, ovarian fluid may be best considered a modified plasma, containing a variety of components such as sugars, hormones, enzymes, proteins and inorganic ions, that appear to have specific functions [6], [7], [9], [11].

Studies in a variety of species indicate that ovarian fluid is likely to have several important biological functions, most notably in reproduction [1], [10], [12]–[16]. For instance, when rainbow trout (*Oncorhynchus mykiss*) ovulated eggs were stored in ovarian fluid for three days, they maintained fertility and developmental competence in contrast to those stored in trout artificial medium [10], [17]. Furthermore, fish often vary widely in egg quality and a female can spawn different clutches of eggs that may have different fertilisability [18]. These differences may be an egg-intrinsic quality of the female, and/or it may represent some qualitative differences in the composition of the ovarian fluid. For example, protein concentration and turbidity of ovarian fluid can be used as indicators to evaluate the fertilising ability of walleye (*Sander vitreus*) eggs [19].

Arguably, one of the most interesting aspects of ovarian fluid biology is its effect on sperm performance. Ovarian fluid in Arctic charr (*Salvelinus alpinus*), chinook salmon, rainbow trout, lake trout (*Salvelinus namaycush*) and brown trout (*Salmo trutta*) is known to have an activating effect on sperm swimming speed, percent motility, and duration of sperm motility [12]–[16], [20]–[24] compared to activation in fresh water [2], [7], [11], [12], [25] or to very dilute ovarian fluid [26], [27]. Though even 10% ovarian fluid has been shown to result in a 70% increase in sperm motility in lake trout [22]. In particular, increased swimming speed is likely biologically significant because sperm swimming speed is recognized as the key determinant of male fertilisation success under conditions of sperm competition in a variety of species, including chinook salmon [21], [28]–[33], but see [34], [35]. In addition, ovarian fluid has been shown to promote fertilisation by conspecific sperm in Atlantic salmon (*Salmo salar*) and brown trout, potentially limiting the risks of genetic incompatibility through inter-specific hybridization [3]. This finding suggests that there is a genetic, species-specific signature within ovarian fluid that helps to ensure male and female compatibility. Moreover, this study demonstrates that ovarian fluid can mediate the selection of gametes from conspecific males at just 1% ovarian fluid concentration; a finding that is significant, since it suggests that sperm selection can occur despite dilution by turbulent river water at spawning sites.

Emerging evidence suggests that ovarian fluid also affects fertilisation success in non-salmonid fishes; in the internally-fertilising guppy (*Poecilia reticulata*), ovarian fluid enhanced the sperm velocity of unrelated males in comparison to related males [36], leading the authors to suggest this might be a mechanism to ward against inbreeding. In contrast, ovarian fluid enhanced sperm performance of related male lake trout [21], prompting speculation that sperm selection of specific genotypes may be important.

Despite the increasing recognition that ovarian fluid may be a potent factor influencing sperm performance and thus, male fertilisation outcomes under conditions of sperm competition, little information is available on its protein composition and how this might vary within a species (but see [37] for a recent study in rainbow trout). Here, we sought to determine whether the protein composition of ovarian fluids differed among female chinook salmon. We subsequently characterized the ovarian fluid using mass spectrometry-based protein analysis and screened sequenced peptides against Mascot and Sequest databases to analyze its proteomic profile. The results were then organized by respective biological process using gene ontology pathway prediction.

## Materials and Methods

### Study species

The chinook salmon used in this experiment were descendants of juvenile fish collected from the major chinook salmon-producing rivers and several isolated land-locked populations on the central South Island of New Zealand [1]. They were acquired from a hatchery-reared population at the National Institute of Water and Atmospheric Research (NIWA) Silverstream Hatchery, Canterbury, New Zealand during the 2004 and 2005 spawning season. All animals were collected, maintained, and culled in accordance with permissions issued by the Animal Ethics Committee for the University of Canterbury, New Zealand.

#### 2.2 Collection of ovarian fluid

Mature three-year-old female salmon were captured from the raceways during the 2004 (n = 15) and 2005 (n = 10) spawning seasons (April–May). Females were checked daily for ovulation (recognized by round and soft abdomens) and fish whose eggs had recently ovulated were killed with a single blow to the head and fish bled by severing the caudal vein. The fish were then cut open with a single cut down the abdomen and their eggs were expelled into a dry dish [2]. Ovarian fluid was gradually retrieved from the egg batch with a pipette, care being taken to avoid any contamination with blood, transferred into screw cap tubes [2], frozen immediately and stored at −80°C.

### 1D SDS-PAGE gel electrophoresis

**Figure 1:**
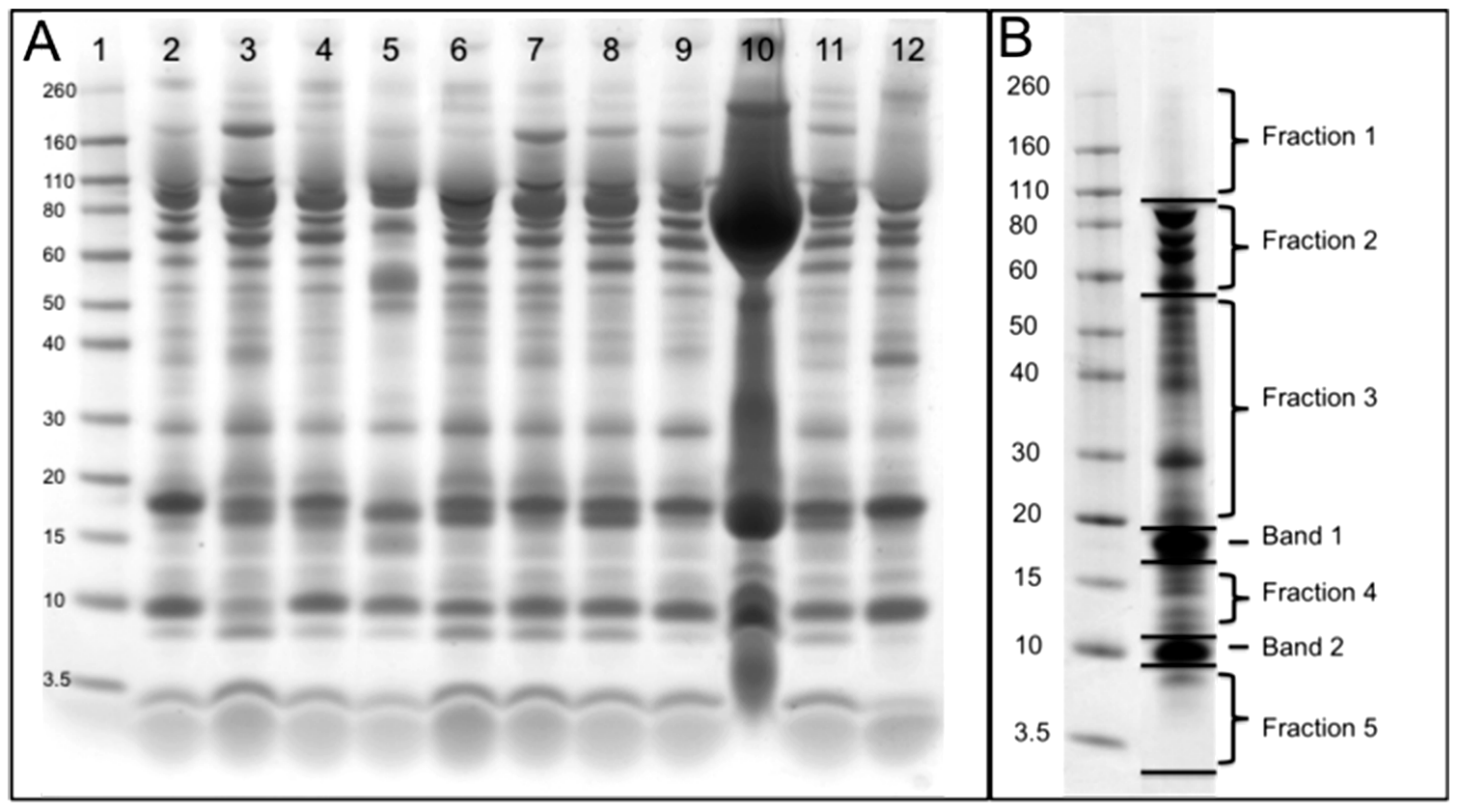
One-dimensional SDS-PAGE profiles of chinook salmon ovarian fluid. A) Comparison of profiles for a representative subset (n = 11) of the 25 ovarian fluids sampled. Lane 1 contains the standard (260 - 3.5 kDa; Invitrogen). Lanes 2–12 contain ovarian fluid samples. Stained with Simply Blue (Invitrogen). 1D-SDS PAGE run conditions were 160 V, 140 mA, 35 min. **B**) Pooled sample of ovarian fluid from 20 females, detailing fractions and bands that were excised for LC-MS/MS. Stained with conventional Coomassie. 1D-SDS PAGE run conditions were 160 V, 140 mA, 35 min.

To examine general differences in protein composition we undertook 1D SDS-PAGE on ovarian fluids from 25 salmon (see Fig. 1A for subset). Electrophoresis was performed using a NuPAGE Novex Bis-Tris Mini Gel Kit (Life Technologies, New Zealand). Briefly, 3 µl of ovarian fluid was loaded onto a 1-mm SDS-containing gel (4–12% polyacrylamide gradient) and run at 120 V for 35–45 minutes after which it was stained with Simply Blue (Life Technologies, New Zealand).

### Sample preparation

Based on initial 1D gels, equal volumes of ovarian fluid from each of 20 females were combined and mixed (we omitted 5 females which showed unusually strong protein bands indicating contamination with albumin and other plasma proteins). From this mixture, a 3 µl aliquot was run on SDS-PAGE and stained with homemade colloidal Coomassie [38]. Five regions (up to 10 kDa, 10–18 kDa, 20–55 kDa, 55–90 kDa, and 90–260 kDa) were excised from the gel (Fig. 1B) and subjected to in-gel digestion with trypsin [39] using a robotic workstation for automated protein digestion (DigestPro Msi, Intavis AG, Cologne, Germany). Two prominent bands (major bands at 10 kDa and 18–20 kDa) were also excised to avoid interference of high abundant proteins. Eluted peptides were dried using a centrifugal concentrator (Savant Speed Vac SC 100; Savant, France).

### Liquid-chromatography mass spectrometry (LC-MS/MS) of tryptic peptides

Samples were re-solubilized in 5% [v/v] acetonitrile, 0.2% [v/v] formic acid in water and injected into an Ultimate 3000 nano-flow uHPLC-System (Dionex Co, Thermo Scientific, San Jose, CA) that was in-line coupled to the nanospray source of an LTQ-Orbitrap XL hybrid mass spectrometer (Thermo Scientific, San Jose, CA). Peptides were separated on an in-house packed emitter-tip column (75 µm ID PicoTip fused silica tubing (New Objectives, Woburn, MA) filled with C-18 material (5 µm bead size) to a length of 12 cm) and eluted by a 35 min. gradient starting at 5% [v/v] acetonitrile, 0.2% [v/v] formic acid and finishing at 80% [v/v] acetonitrile, 0.2% [v/v] formic acid in water at a flow rate of 500 nl/min.

### Typical instrument setting for the LTQ-Orbitrap

Full MS in a mass range between m/z 400–2000 was performed in the Orbitrap mass analyzer with a resolution of 60,000 at m/z 400 and an AGC target of 5e5 for each of the gel fractions. Preview mode for FTMS master scan was enabled to generate precursor mass lists. The strongest 5 signals were selected for CID (collision-induced dissociation)-MS/MS in the LTQ ion trap at a normalized collision energy of 35% using an AGC target of 2e4 and one microscan. Dynamic exclusion was enabled with 2 repeat counts during 30 s and an exclusion period of 180 s. Exclusion mass width was set to 0.01.

### Data analysis

Raw spectra were processed through the Proteome Discoverer software (Thermo Scientific, San Jose, California, USA) using default settings. For protein identification, MS/MS spectra were first searched against a Teleostei subset of the NCBI nr amino acid sequence database (214337 entries, downloaded July 2012) using three different search engines, SEQUEST (Thermo Scientific), Mascot (www.matrixscience.com) and MS Amanda (http://ms.imp.ac.at/?goto=msamanda). The searches were set up for tryptic cleavages with a maximum of three missed cleavage sites and carboxyamidomethyl cysteine, oxidized methionine and deamidated asparagine were included as variable modifications. The precursor mass tolerance threshold was 10 ppm and the maximum fragment mass error 0.8 Da.

We have used different search programs and a strict statistic to filter out false discoveries. The Discovery Rate (FDR) was estimated using the Percolator algorithm (http://per-colator.com). Peptide hits were filtered for a FDR of q<0.01. In addition to the FDR filter, threshold score filters for each search engine were applied to eliminate very low scoring peptide hits that may have passed the Percolator FDR filter. The score thresholds were estimated by manual examination of very low scoring hits at a FDR of q<0.01. The following score thresholds for positive peptide identification were applied: Mascot peptide score of >20, MS Amanda score >100 and charge state (z)-dependent SEQUEST cross correlation scores (XCorr) of 2.25 (z = 2), 2.5 (z = 3), 3.0 (z = 4 or z = 5), 3.5 for all other charge states). All spectra that were not assigned to any sequence or did not pass the filters were searched again against all EST nucleotide sequences available for Salmonidae in the NCBI nr database (835001 entries, downloaded January 2013) using the Mascot search engine. Search settings and filters were as described above. All searches were combined using the Proteome Discovery software. Only proteins/protein groups that were identified by two or more independent peptide hits and by at least two search engines (only for identifications from the Teleostei amino acid sequence database) were accepted as true positive hits. Identified proteins were assigned to biological processes (Gene Ontology annotation) using the GOanna search tool at the AgBase server (http://www.agbase.msstate.edu/cgi-bin/tools/GOanna.cgi).

## Results

Analysis of ovarian fluid samples from 25 mature females using one dimensional SDS-PAGE showed significant variability in the abundance of proteins, as well as variation in the absence or presence of particular protein bands. A representative assortment (n = 11) of the 25 individuals is presented in Figure 1A. The samples loaded in lanes 3 and 7 showed particularly strong protein bands around 160 kDa, while the sample in lane 5 has a band at approximately 55 kDa that is not present in other lanes. Across the samples, we also observed significant variability in the number of protein bands observed in the region spanning 40–110 kDa. Note that the sample in lane 10 appears to be contaminated with albumin, hence this sample was not included in the pooled aliquot for LC-MS/MS. Likewise, samples in lane 3 and 12 were not included, in order to avoid over-representation of particular samples in the pooled aliquot.

In the twenty samples that we pooled (Fig. 1B) for liquid-chromatography mass spectrometry (*LC-MS/MS)* 174 proteins were identified (Table S1 and S2). The pathway-based enrichment analysis classified proteins according to “biological process” (Table S3). Six GO categories (*response to chemical stimulus, response to hypoxia, response to estradiol, response to bacterium, somitogenesis, and epiboly involved in gastrulation with mouth forming second*) contained six or more proteins (Table 1).

**Table 1:** Predominant biological pathways identified in chinook salmon ovarian fluid. Gene Ontology annotations (biological process) based on the AgBase v2.00 database.

GO Pathway Category	Number of proteins in category
Response to chemical stimulus	14
Response to hypoxia	12
Response to estradiol stimulus	8
Response to bacterium	8
Somitogenesis	9
Epiboly involved in gastrulation with mouth forming second	6

## Discussion

As anticipated, the SDS-PAGE banding patterns, which reflect the protein composition of the ovarian fluid, differed markedly among female chinook salmon. This finding is important because the composition of ovarian fluid is believed to interact with the sperm swimming speed, and hence, fertilisation outcomes, in this species [1] and in others [3], [21]. For instance, sperm swimming speed, path trajectory and longevity differ significantly among males, at least in salmon, and are all affected by the ovarian fluid from different females, suggesting that variation in the composition of this fluid interacts with sperm performance traits [1]. The most likely explanation for these interaction effects on sperm traits is the presence of some form of molecular interaction (see below), whereby components of female ovarian fluid differentially enhance the function of sperm from different males. Such a mechanism may act to promote the success of favored male genotypes in a post-copulatory form of sexual selection known as cryptic female choice [1], [40].

Prior work from our team on chinook salmon found that concentrations of inorganic components in ovarian fluid vary and affect sperm function (e.g., Ca^2+^and Mg^2+^), [2], but that these factors were unable to explain the strong male×female interaction observed [1]. Hence, the most likely contenders to mediate such an interactive effect are proteins. Rapidly evolving female and male reproductive proteins have been identified as mediators for fertilisation at a gamete level in several taxa including rodents, *Drosophila*, sea urchins and seastars [41]–[44]. For example, in the frog, *Crinia georgiana*, a similar pattern emerges, and sperm motility is affected by egg jelly leading to female and male×female interactive effects on fertilisation outcomes [45]. An egg jelly protein, allurin, that has sperm chemo-attractant properties [46], may be an important contributor to this effect. Other sperm attraction and sperm-activating proteins have been identified in broadcast spawning marine invertebrates [42], [43], [47], [48], and the Pacific herring (*Clupea pallasii*), in which two sperm-activating proteins were found [49]–[52]. Further, recent work [3] suggests that the straightening of the sperm trajectory, observed when activating sperm in ovarian fluid, but not in water, is behaviour that is consistent with chemotaxis into and up a biochemical concentration gradient. The authors suggest that since ovarian fluid should be most concentrated in the micropyle, then chemotaxis provides a mechanism to allow species-specific signaling between ovarian fluid and sperm, via reproductive proteins [3]. Unfortunately, in this study we found no homology among the 174 proteins we identified in the pooled sample of ovarian fluids from 20 random females to known sperm attraction and sperm activating peptides. However, such proteins are often under strong selection and evolve quickly thus finding homologues might well be challenging [42].

Our work shows that salmon ovarian fluid has a complex and variable composition, and it is likely that the proteins that make up this fluid originate from a suite of sources, such as the extracellular matrix, the serum, red blood cells, the immune system and the egg. Plasma contamination is a possibility given the way ovarian fluid is sampled (i.e., by cutting open the abdomen to expel eggs – though fish are bled beforehand) and future work should include quantitative comparison of biological replicates of individual OF samples, to distinguish between ovarian fluid proteins and plasma contamination. While we only analysed a representative pool of 20 female's ovarian fluids, further work is needed to quantitatively compare the protein profiles of ovarian fluid isolated from different females.

Several of the proteins we identified in chinook salmon were also found in a recent study on its close relative the rainbow trout [37], including markers of oocyte quality (e.g., apolipoprotein A-I-1,several forms of vitellogenins, and mannose-binding lectin) and immune function (e.g., complement components C3 and 4, lysozyme C-11, precerebellin-like protein). While this recent work identified 54 proteins, compared to the 174 we identified in chinook salmon, they found a number of proteins in rainbow trout OF that were not observed in the chinook salmon OF samples we analysed (e.g., apolipoprotein E precursor, various other complement components, precerebellin-like protein precursor, vitelline envelope protein gamma precursor; [37]).

Within the chinook salmon dataset, a number of proteins were assigned to the GO pathway *response to chemical stimulus* (Table 1). Of these, the complement components, produced by the immune system, particularly stand out as being potentially important in affecting sperm performance traits. The complement system is a major part of innate and adaptive immunity and these proteins are maternally transferred in rainbow trout [53]. Complement components are important to non-immunological processes as well. For instance, complement C3 in the human female reproductive tract is thought to function in the clearance of dead or dysfunctional sperm, and to also be important in a series of events that lead up to fertilisation [54]. However, C3 complement is a common plasma protein and may simply represent carryover from plasma in the ovarian fluid. But, additional complement components (C6, C7-2, C9) were identified in trout ovarian fluid [37] and complement proteins are further abundant in the seminal fluid of rainbow trout [55]. Whether the complement proteins play a role in sperm motility in chinook salmon is unclear and requires a quantitative analysis, but certainly warrants further exploration.

Several other noteworthy proteins were found in the ovarian fluid of chinook salmon that are known to be important for reproductive functions, but could also be mediators of the effects previously observed between ovarian fluid and sperm in this system. For example, numerous forms of vitellogenin were identified in chinook salmon ovarian fluid, similar to that found in rainbow trout [10], [37] and Persian sturgeon (*Acipenser persicus*) ova [56]. Vitellogenin polymorphism could be important in mediating the sperm motility effects observed in chinook salmon. For this to be the case, however, insight into the appearance of vitellogenin into the ovarian fluid (incidental cell rupture (c.f., [10]) or intentional transfer from the serum) is desirable.

Cathepsin D, another potential modulator of sperm performance, is an aspartic protease with diverse functions. Early work linked it to non-specific protein degradation in lysosomes [57] and to poor quality eggs in sea bass (*Dicentrachus labrax*) and sea bream (*Sparus aurata*) [10], [58], [59], suggesting it may be a marker of egg quality and originates from eggs rather than ovarian fluid. However, the ability of cathepsin D to cleave structural and functional proteins and peptides has also seen it implicated in numerous other physiological functions, including metabolic degradation of intracellular proteins, activation and degradation of polypeptide hormones and growth factors, activation of enzymatic precursors, processing of enzyme activators and inhibitors, brain antigen processing and regulation of apoptosis (reviewed in [57].

While there is no direct evidence of cathepsin D influencing sperm function in fish, there is accumulating data that suggest cathepsin D may mediate aspects of sperm function in other systems. Cathepsin D is detected on the sperm surface in humans and undergoes modification to an active form during capacitation [60], in which process it likely plays an important role in the conversion of proacrosine to acrosin [61]. Cathepsin D concentrations in cauda epididymis fluid also correlate strongly with fertility scores of cattle [62], but cathepsin D concentrations in seminal plasma showed no clear relationship with sperm count and morphology in humans [63]. Given its roles in capacitation and its associations with heightened fertility, albeit in mammalian systems, sperm motility is the most likely sperm trait linked to fertility outcomes that is affected by cathepsin D. While the amount of cathepsin D identified in the ovarian fluid pool in both this study and rainbow trout (Nynca et al., 2014) is low and while cathepsin D may be derived from eggs rather than ovarian fluid, the possibility that cathepsin D levels may have positive effects on sperm activation and enhanced sperm movement is worthy of further exploration.

Mechanistically, we consider proteins involved in immune function and/or chemical signaling to be promising candidates for modulating sperm performance. Examples abound of molecular mechanisms that evolved in other biological systems to separate ‘self’ from ‘non-self’, yielding a glimpse of how ovarian fluid could signal ‘incompatibility’ (genetic similarity) to sperm – these examples tend to centre around the expression of (highly) polymorphic genes, which in some instances mediate their effects as secreted signaling molecules; thus, many flowering plants have mechanisms in place to prevent in-breeding through self-fertilisation. Three notably different mechanisms appear to have evolved and have been elegantly illustrated [64]; in some plants, cells of the stigma express a membrane receptor kinase (female determinant; product of a polymorphic gene) that binds a small protein (male determinant; product of a polymorphic gene) released from the pollen upon pollination. If the pollen is from ‘self’, then the receptor kinase is activated and the pollen rejected. Another group of plants secretes a particular RNase (highly polymorphic) from the style, part of the female sex organ, into the extracellular matrix. This RNase enters the pollen and interacts with a male determinant to bring about RNA degradation if ‘self’ or to be degraded by proteolysis if ‘non-self’. A third mechanism is based around the release of a variable protein by the stigma (female) that interacts with a receptor on the pollen to activate an intracellular pathway leading to cell death [64].

Filamentous fungi also evolved incompatibility genes, so-called heterokaryon (*het*) genes, and a difference between individuals among any of the 5–11 *het* loci, expressed on the plasma membrane can lead to fusion avoidance [65]. Precisely which molecular events lead to fusion avoidance and death of contacting fungal hyphae is not currently known, but it has been suggested that a conformational change, in the case of HET-C2, may be important to initiate an apoptotic response [66].

Beyond self-incompatibility, the difficulties associated with crossing closely related plant species (e.g., poor pollen tube growth and failure of pollen grains to germinate) have long been recognized [67], [68]. Conspecific pollen may have faster pollen tube growth, and have pollen tubes that are less likely to burst prematurely and more likely to negotiate the micropyle of the egg [69]. Variation in reproductive proteins may drive rapid divergence and species specificity in post-mating mechanisms [70], ensuring conspecific compatibility. Such reproductive proteins may be analogous to those found in animals [41], [42], [71], [72]. Selection on gamete-recognition proteins is also known to influence reproductive success within a species. For example, in sea urchins, fertilisation is controlled by a highly polymorphic gamete recognition protein, bindin [73]. Studies have demonstrated that there is selection for rare bindin genotypes in high density populations where sperm competition is common and selection for common bindin genotypes in low density populations where individuals are faced with sperm limitation [73].

Finally, non-random sperm selection for like and dislike MHC class I and II genotypes, respectively, has been documented in chinook [74] and Atlantic salmon [75], [76]. MHC dependent mate choice has been observed across a range of taxa [75], [76], and the pervading view is that such genotypic selection operates to promote offspring MHC genetic diversity, increasing offspring fitness against pathogenic attack, and/or as a mechanism for differentiating kin, either to avoid inbreeding, or to avoid hybridisation and preserve local adaptations [75], [76]. One intriguing possibility is that ovarian fluid somehow mediates MHC dependent discrimination, with some prior work suggestive of MHC selectivity at the gamete level [75], [76]. However, the evidence for MHC dependent sperm selection remains equivocal and to date no mechanism has been identified.

In conclusion, this study represents the first application of proteomics for the characterization of chinook salmon ovarian fluid and allowed the identification of 174 different proteins. Unfortunately, the genome of salmon is not yet fully sequenced, so there are many more proteins that remained unidentified; regardless, the current data set provides a resource that can contribute to furthering our understanding of factors that influence successful egg production and fertilisation in salmonids and other species. Analysis of the ovarian fluid proteomic profile in female chinook salmon may eventually reveal the identity of sperm-activating proteins that bring about differential effects on sperm swimming speed. Future work will focus on quantitative differences between females with known sperm motility patterns.

## Supporting Information

Table S1 **Non-redundant list of significant protein identifications.** Accession number (Accession) and description (protein group) from the Teleostei amino acid sequence database (Db: aa) or the est sequence database (Db: est). The number of different peptides (identified peptides) and the number of spectra acquired and identified per protein (Peptide spectrum match) as well as the average peak area of the three strongest peptide hits per protein (Area under the curve) can be used as a rough estimation of relative abundance. The total protein score is given for each search engine (SEQUEST, Amanda and Mascot protein score). Note the est database was only searched with Mascot and there is no area under the curve calculation available for this search. The molecular weight (MW) and isoelectric point (p*I*) of identified proteins have been calculated based on the amino acid sequence database entry. EST entries were not considered for MW and p*I* calculation.
(XLSX)

Table S2 **Significant protein identifications for bands 1 and 2 excised from 1D SDS-PAGE gel (see** **Figure 1B** **).** Accession number (Accession) and description (protein group) from the Teleostei amino acid sequence database (Db: aa) or the est sequence database (Db: est).
(XLSX)

Table S3 **List of significant protein identifications with gene ontology annotations based on the AgBase v2.00 database.**
(XLSX)
